# The Use of a Computerized Cognitive Assessment to Improve the Efficiency of Primary Care Referrals to Memory Services: Protocol for the Accelerating Dementia Pathway Technologies (ADePT) Study

**DOI:** 10.2196/34475

**Published:** 2022-01-27

**Authors:** Chris Kalafatis, Mohammad Hadi Modarres, Panos Apostolou, Naji Tabet, Seyed-Mahdi Khaligh-Razavi

**Affiliations:** 1 Cognetivity Neurosciences Ltd London United Kingdom; 2 South London & Maudsley NHS Foundation Trust London United Kingdom; 3 Department of Old Age Psychiatry King's College London London United Kingdom; 4 Dementia Research Unit Sussex Partnership NHS Foundation Trust West Sussex United Kingdom; 5 Centre for Dementia Studies Brighton and Sussex Medical School Brighton United Kingdom; 6 Department of Stem Cells and Developmental Biology Cell Science Research Center Royan Institute for Stem Cell Biology and Technology Tehran Iran

**Keywords:** primary health care, general practice, dementia, cognitive assessment, artificial intelligence, early diagnosis, cognition, assessment, efficiency, diagnosis, COVID-19, memory, mental health, impairment, screening, detection, efficiency

## Abstract

**Background:**

Existing primary care cognitive assessment tools are crude or time-consuming screening instruments which can only detect cognitive impairment when it is well established. Due to the COVID-19 pandemic, memory services have adapted to the new environment by moving to remote patient assessments to continue meeting service user demand. However, the remote use of cognitive assessments has been variable while there has been scant evaluation of the outcome of such a change in clinical practice. Emerging research in remote memory clinics has highlighted computerized cognitive tests, such as the Integrated Cognitive Assessment (ICA), as prominent candidates for adoption in clinical practice both during the pandemic and for post-COVID-19 implementation as part of health care innovation.

**Objective:**

The aim of the Accelerating Dementia Pathway Technologies (ADePT) study is to develop a real-world evidence basis to support the adoption of ICA as an inexpensive screening tool for the detection of cognitive impairment to improve the efficiency of the dementia care pathway.

**Methods:**

Patients who have been referred to a memory clinic by a general practitioner (GP) are recruited. Participants complete the ICA either at home or in the clinic along with medical history and usability questionnaires. The GP referral and ICA outcome are compared with the specialist diagnosis obtained at the memory clinic. The clinical outcomes as well as National Health Service reference costing data will be used to assess the potential health and economic benefits of the use of the ICA in the dementia diagnosis pathway.

**Results:**

The ADePT study was funded in January 2020 by Innovate UK (Project Number 105837). As of September 2021, 86 participants have been recruited in the study, with 23 participants also completing a retest visit. Initially, the study was designed for in-person visits at the memory clinic; however, in light of the COVID-19 pandemic, the study was amended to allow remote as well as face-to-face visits. The study was also expanded from a single site to 4 sites in the United Kingdom. We expect results to be published by the second quarter of 2022.

**Conclusions:**

The ADePT study aims to improve the efficiency of the dementia care pathway at its very beginning and supports systems integration at the intersection between primary and secondary care. The introduction of a standardized, self-administered, digital assessment tool for the timely detection of neurodegeneration as part of a decision support system that can signpost accordingly can reduce unnecessary referrals, service backlog, and assessment variability.

**Trial Registration:**

ISRCTN 16596456; https://www.isrctn.com/ISRCTN16596456

**International Registered Report Identifier (IRRID):**

DERR1-10.2196/34475

## Introduction

Worldwide, national dementia strategies emphasize the need for improving the diagnostic pathway at the point of primary care toward timely diagnosis. Currently, general practitioner (GP) clinical judgement of cognitive impairment is the basis of referral initiation to specialist services. Existing primary care cognitive assessment tools (eg, the General Practitioner Assessment of Cognition [GPCOG], the Mini-Cog, and the Six-Item Cognitive Impairment Test [6CIT]), are crude or time-consuming screening instruments which can only detect cognitive impairment when it is well established. Dementia is difficult to diagnose; in a study concerning false positive diagnoses, 60% of GPs misdiagnosed dementia [1]. More detailed tests deployed in secondary care are expensive and often physically and psychologically intrusive for the patient (eg, lumbar puncture). As a result, many false positives are identified in referred patients. A key limitation of existing screening tests is the lack of robust evidence to support them; few have been well validated in the populations for which they are intended.

**Figure 1:**
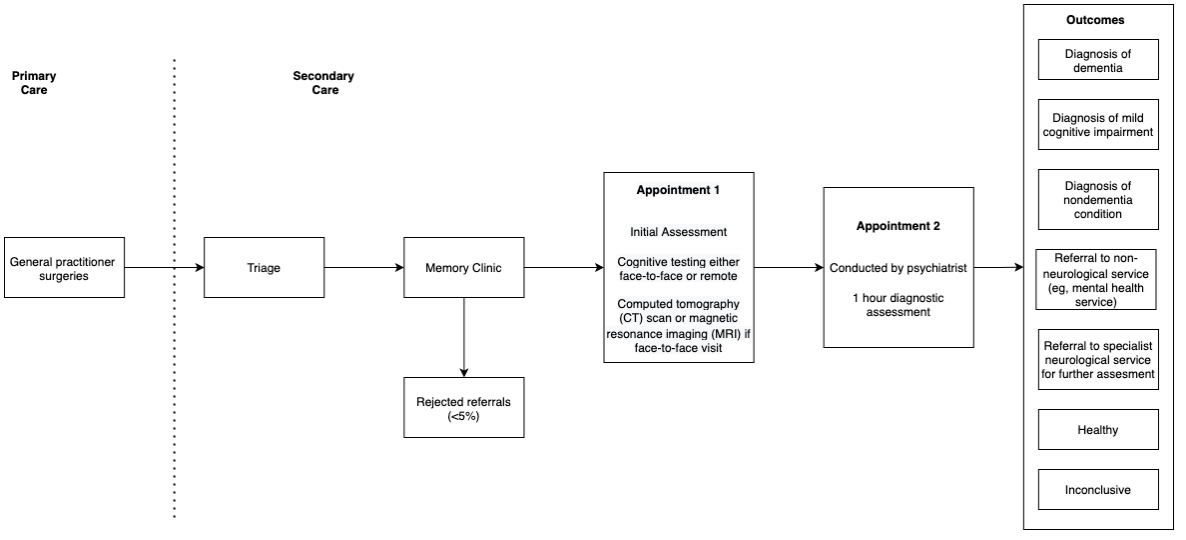
Dementia diagnostic pathway for patients.

Figure 1 demonstrates the dementia diagnostic pathway for patients. Patients who are referred by their GP are triaged. At the memory clinic, patients undergo 2 appointments; the first is typically conducted by a nurse and involves administration of a cognitive assessment. At the second appointment (the diagnostic clinic visit), conducted by a dementia medical specialist, the patient receives the outcome of the assessment (see “Outcomes” within Figure 1 for examples of typical outcomes).

The COVID-19 pandemic has effectively brought clinical practice in the memory services to a standstill. Nationally, memory services have adapted to the new environment by moving to remote patient assessments to continue meeting service user demand while reducing viral transmission [2]. However, the remote use of cognitive assessments has been variable, while there has been scant evaluation of the outcome of such a change in clinical practice [3]. Emerging research in remote memory clinics has highlighted computerized cognitive tests, such as the Integrated Cognitive Assessment (ICA), as prominent candidates for adoption in clinical practice both during the pandemic and for post-COVID-19 implementation as part of health care innovation [4].

The ICA is a 5-minute computerized cognitive test based on a rapid categorization task that employs an artificial intelligence model to improve its accuracy in detecting cognitive impairment [5]. The ICA is self-administered and independent of language [6,7]. The value proposition of the ICA is that an accurate and sensitive tool for diagnosis will streamline the diagnosis of dementia by reducing false positive results from GP referrals and, therefore, minimizing the need for further, expensive and time-consuming assessments.

In order to address this challenge, we initiated the Accelerating Dementia Pathway Technologies (ADePT) study. The intention of conducting this study is to develop a real-world evidence basis to support the adoption of ICA as an inexpensive screening tool for the detection of cognitive impairment to improve the efficiency of the dementia care pathway.

The ADePT study is an ongoing multicenter real-world evidence study. The objective of ADePT is to deliver real-world evidence on practices and the economic case for ICA adoption in memory clinics for the assessment of cognitive impairment associated with dementia, Alzheimer Disease (AD), mild cognitive impairment (MCI), and similar diseases, including the assessment of preferred business models by comparing the accuracy of GP referrals against the ICA.

## Methods

### Ethics Approval

Health Research Authority and Health and Care Research Wales approval for this study was obtained in February 2020. The study is registered in the ISRCTN Registry (ISRCTN16596456).

### Study Design

All participants are recruited among attendees at the National Health Service (NHS) memory services at the point of referral by their GP. The participants who do not have a formal diagnosis of a neurodegenerative disease are triaged as per usual clinical practice and are asked to complete the ICA in parallel with the diagnostic assessment. The aim of the clinical work package is to recruit 140 participants into the study.

The main study inclusion criterion is referral to the memory clinic by a GP. Patients recruited must be 55 to 90 years old. Potential participants must also be fully informed of and understand the objectives, procedures, and possible benefits and risks of the study and have the capacity to provide written consent.

Subjects that meet the following criteria will be excluded from the study cohort:

- Lack of capacity to consent to participation in this study
- Upper limb arthropathy or motor dysfunction that limits the use of a tablet computer
- Visual impairment severe enough to limit the use of a tablet computer
- Known diagnosis of dementia
- Already receiving cholinesterase inhibitors and/or Memantine

### Study Procedures

Participants enrolled in the study will be required to attend 1 visit at a designated memory clinic or remotely at their home (Assessment Visit 1 [AV1]). Participants will be asked to complete the ICA. Prior to taking the ICA, participants will also be requested to view a short training video to assist them in completing the task successfully. After taking the ICA, patients will complete the following short questionnaires:

- Inquiry on stimulants, fatigue, and sleep: A questionnaire that assesses the participant’s overall state. Questions revolve around recent intake of stimulants (eg, coffee or alcohol), sleep quality, energy levels, and mood. The questionnaire is used in conjunction with the ICA to determine whether any of these factors have had an impact on ICA performance.
- ICA Usability Questionnaire: A questionnaire that assesses the participant’s views on their experience with the test to receive acceptability and usability feedback for the ICA.
- Cognitive Health Questionnaire: A questionnaire that assesses the participant’s history of activities of daily living and physical and mental health comorbidities. The questions should ideally be answered by the informant (study partner) if available or by the participant if an informant is not present. The questionnaire is used in conjunction with the ICA to determine whether cognitive impairment detected by the ICA is due to MCI/dementia or other organic and/or treatable conditions.

Lastly, a brief medical history of the participants via electronic health care records will be obtained, mainly focusing on any cognitive tests that have been taken by the participants.

**Figure 2:**
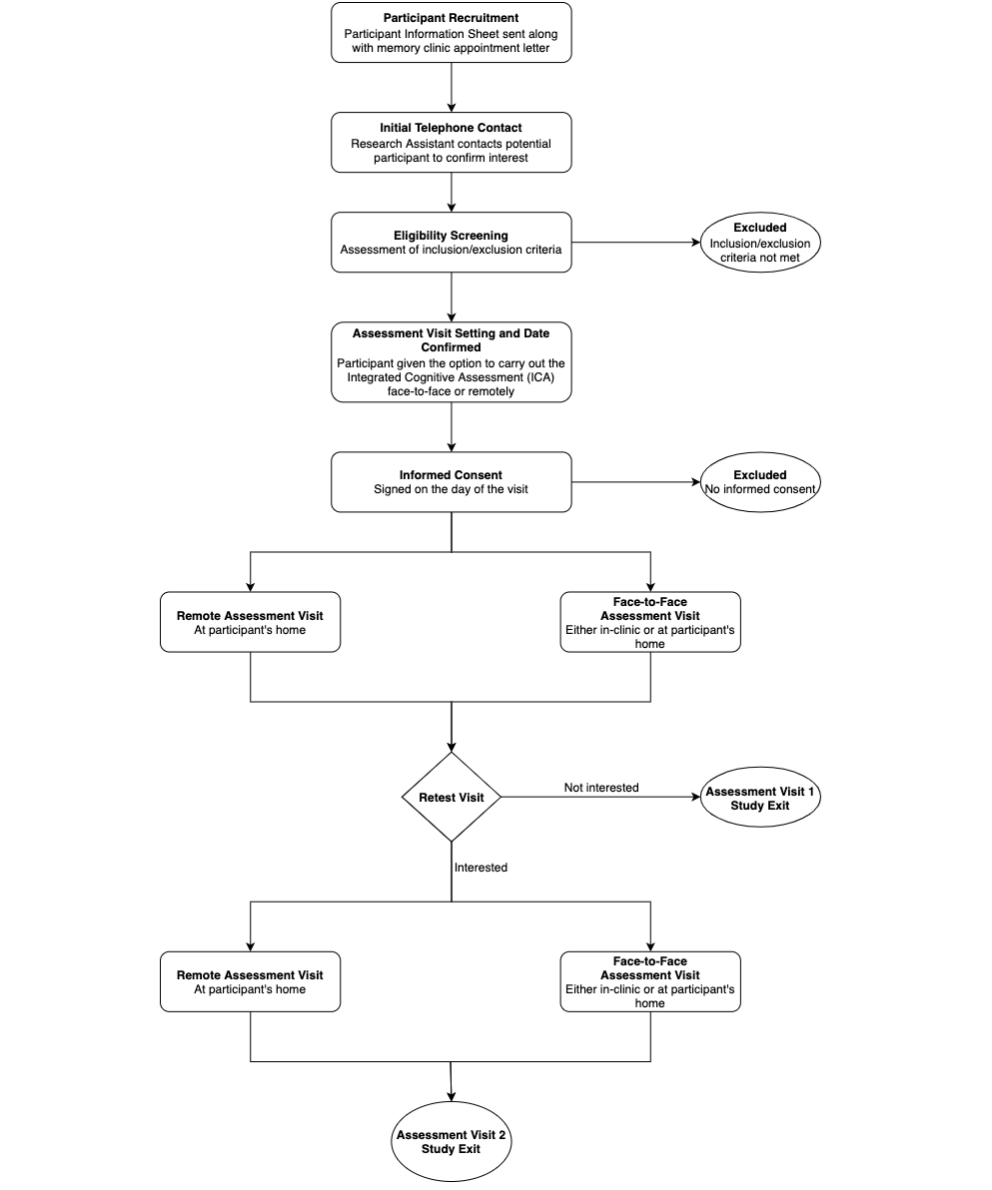
Accelerating Dementia Pathway Technologies (ADePT) study participant pathway.

Participants will also be given the option to carry out a retest visit (Assessment Visit 2 [AV2]) whereby they are again given the chance to take the ICA test either remotely or face-to-face, complete a usability questionnaire, and respond to inquiries on stimulants, fatigue, and sleep. The overall study pathway for participants is detailed at a high level within Figure 2.

### Data Management

The primary data sources are the Castor electronic data capture (EDC) system and the ICA portal. Castor EDC will be used to report all protocol-required information for each participant in the form of an electronic case report form. The participants of the study are not identified by name or initials on the electronic case report form or any other study documents to be collected by Cognetivity Neurosciences Ltd, but will be identified by a participant ID number. Data entry is performed by the researchers in the investigator sites, while data source verification is performed by the sponsor’s clinical research associate.

The ICA portal is a secure portal where ICA results are uploaded. In addition, the participant’s ID number and demographic details are also uploaded to the ICA portal, which allows linking of the ICA data to the Castor EDC data. Data entered in the iPad (Apple Inc) do not undergo source data verification; however, the ID field and key fields which are common between the ICA and EDC undergo a data check by the data manager and queries are raised in case of discrepancy.

The secondary data source is source data, which includes any information in original records and certified copies of original records of participants, including medical history records as well as worksheets, which are usually used in order to record protocol-required information during the assessment of each participant (eg, usability questionnaires) prior to inputting such data into Castor EDC.

Data linkage and processing then takes place, from which metrics and data sets for analysis are generated. Such metrics include, but are not limited to, demographic breakdown (to track the distribution of age, years of education, and gender), a spreadsheet of key data fields used by the Medical Monitor to review data for a patient as well as site metrics on recruitment, queries and protocol deviations, lock and sign off, study exit, and adverse events. 

### Statistical Analysis

For the purposes of these analyses, patients referred to the memory clinic are divided into the following 3 groups, based on their memory clinic outcome: (A) those who receive a diagnosis of MCI or dementia, (B) those who are identified as healthy or receive a diagnosis of a brain or mental disorder other than MCI or dementia, and (C) those who receive an inconclusive diagnosis.

Participants with an inconclusive outcome after the memory clinic assessment are excluded from further analysis. 

Participants in group A are counted as correct GP referrals. Participants in group B are counted as unnecessary or incorrect referrals.

#### Comparison with Specialist Diagnosis of MCI/Dementia

The metrics for GP referrals that will be calculated are the following:

- Total number of patients referred by GPs=A+B+C
- Proportion of necessary GP referrals (excluding inconclusive)=A/(A+B)
- Proportion of unnecessary GP referrals (excluding inconclusive)=B/(A+B)

Likewise, the following complementary metrics for the ICA will be calculated:

- Total number of patients the ICA would have referred 
- Proportion of patients correctly referred by the ICA 
- Proportion of patients incorrectly referred by the ICA 
- Proportion of patients correctly not referred by the ICA 
- Proportion of patients incorrectly not referred by the ICA 

In a secondary outcome analysis, we will compare with specialist diagnosis of all types of cognitive impairment (those due to MCI, dementia, or other neurological or mental disorders).

#### Test-Retest Analysis

The test-retest reliability of the ICA will be analyzed by the following:

- Calculation of intraclass correlation coefficient to assess test-retest reliability across all participants
- Scatterplot construction and calculation of correlation coefficient between the initial and final assessment for all participants
- Construction of Bland-Altman plots for the initial and final assessment to assess agreement

### Qualitative Data from Usability Questionnaire

Multiple choice responses from participants will be analyzed by calculating the proportion of participants who selected each option. Questions relating to frequency of tablet or mobile phone use will be used to assess familiarity with technology, in particular touch screen devices. The ease of understanding the ICA instructions and level of difficulty of the categorization task will be analyzed by calculating the proportion of participants who reported finding each of these steps very easy, easy, moderately difficult, difficult, or very difficult.

### Procedures to Account for Missing and Spurious Data

Patients with inconclusive outcomes are excluded from our analysis. Other than that, we do not expect any other missing data regarding the calculations needed for primary and secondary outcome measures.

### Health Economic Evaluation

The clinical outcomes described above and data gathered from surveys, in combination with NHS reference costing data, will be used to assess the potential health economic benefits of the use of the ICA in the dementia diagnosis pathway.

The inputs that are actively gathered as part of this study to be used for health economic modelling are the following:

- Comparison of ICA referrals with specialist diagnosis 
- If the participant was referred to another secondary care team

NHS reference costing data (or other literature review) will be used to determine the cost of patient diagnosis considering the cost of the GP appointment and assessments performed at the memory clinic. 

Based on the outcomes in the statistical analysis, we will compare the total costs and time saved if ICA was to be used by the GP for referral or at the entry to memory clinics to triage patients before entering the full diagnostic pathway. 

## Results

The ADePT study was funded in January 2020 by Innovate UK (Project Number 105837). The first patient visit was conducted in November 2020.

As of September 2021, 86 participants have been recruited for the study, with 23 participants also completing a retest visit. Initially, the study was designed for in-person visits at the memory clinic; however, in light of the COVID-19 pandemic, the study was amended to allow remote as well as face-to-face visits.

The study was also expanded from a single site to 4 sites in the United Kingdom, based at the following trusts: Devon Partnership NHS Trust, North Bristol NHS Foundation Trust, Oxford Health NHS Foundation Trust, and Sussex Partnership NHS Foundation Trust.

We expect results to be published by the second quarter of 2022.

## Discussion

In summary, the ADePT project aims to improve the efficiency of the dementia pathway at its very beginning and supports systems integration at the intersection between primary and secondary care. The introduction of a standardized, self-administered, digital assessment tool for the timely detection of neurodegeneration as part of a decision-support system that can signpost accordingly can reduce unnecessary referrals, service backlog, and assessment variability.

Remote assessments in the post-COVID-19 clinical environment are expected to form a core part of frontline service delivery as both services and their service user attitudes change while the use of smartphones and tablet computers is expanding in older adults [8]. Early identification is key as evidenced by the Prime Minister’s Challenge in 2020 [9] and is now corroborated by the advent of novel disease-modifying treatments [10].

We hypothesize that the health economic benefits for such a decision support tool will overshadow the relatively low price of such a proprietary technology compared to pen and paper conventional tests that demand time and expertise most primary care practitioners may not have, in combination with limitations in their validity in prodromal dementia and invariable cultural and interpretation bias.

The main strength of this study is that it accommodates face-to-face and remote assessments as part of day-to-day practice in a memory service setting with minimal disruption to the pathway, while obtaining real-world data on participant experience and the acceptability of such a tool in a rapidly changing technological environment.

In the ADePT study, we investigate GP referrals to memory clinics, where GP colleagues have already made the clinical decision to refer to specialists. As a result, these cases may only be assessed as true or false positives based on prospective diagnosis of neurodegeneration or not.A more complete picture of the quality of primary care cognitive assessment would also require the study of true and false negatives (ie, those patients who were not referred to memory clinics by their GPs). This is a limitation of the study, albeit an unavoidable one as the latter would require multisite recruitment from GP surgeries and prospective participant follow-up which would be impractical. However, we expect that the ADePT results can inform a future longitudinal study in primary care.

To date, research on digital cognitive tests in primary care has been scant. A valid and acceptable tool can improve early diagnosis, provide timely interventions, enhance clinical and health economic outcomes, reduce burden on memory services, and foster interoperability and continuity of care throughout the patient journey in the dementia pathway. A remote assessment tool can also close the clinically important service gap that is the monitoring of disease progression both in already diagnosed patients and those with MCI. Monitoring of the latter patient group is particularly topical as there is no current service provision, as novel treatments are becoming available [10]. The ADePT project aims to provide appropriate evidence to support policy change to shape best practice guidelines in the dementia pathway. Finally, objective and consistent measurement of cognition for at-risk populations will support research and candidate identification in primary care for a disease that continues to take a toll on patients, services, and families.

## Abbreviations

6CIT: Six-Item Cognitive Impairment Test
AD: Alzheimer Disease
ADePT: Accelerating Dementia Pathway Technologies
AV1: Assessment Visit 1
AV2: Assessment Visit 2
EDC: electronic data capture
GP: General practitioner
GPCOG: General Practitioner Assessment of Cognition
ICA: Integrated Cognitive Assessment
MCI: mild cognitive impairment
